# Supplementary material for: Recovery of neural dynamics criticality in personalized whole-brain models of stroke
Source: Nat Commun. 2022 Jun 27;13:3683. doi: 10.1038/s41467-022-30892-6 (PMC9237050; doi:10.1038/s41467-022-30892-6)
Supplement: Supplementary file 2 — Reporting Summary [file 41467_2022_30892_MOESM2_ESM.pdf]

nature portfolio

Corresponding author(s): Rodrigo Pereira Rocha

Last updated by author(s): Apr 14, 2022

Reporting Summary

Nature Portfolio wishes to improve the reproducibility of the work that we publish. This form provides structure for consistency and transparency in reporting. For further information on Nature Portfolio policies, see our Editorial Policies and the Editorial Policy Checklist.

Statistics

For all statistical analyses, confirm that the following items are present in the figure legend, table legend, main text, or Methods section.

n/a	Confirmed	
☐	☒	The exact sample size (n) for each experimental group/condition, given as a discrete number and unit of measurement
☐	☒	A statement on whether measurements were taken from distinct samples or whether the same sample was measured repeatedly
☐	☒	The statistical test(s) used AND whether they are one- or two-sided<br>*Only common tests should be described solely by name; describe more complex techniques in the Methods section.*
☐	☒	A description of all covariates tested
☐	☒	A description of any assumptions or corrections, such as tests of normality and adjustment for multiple comparisons
☐	☒	A full description of the statistical parameters including central tendency (e.g. means) or other basic estimates (e.g. regression coefficient) AND variation (e.g. standard deviation) or associated estimates of uncertainty (e.g. confidence intervals)
☐	☒	For null hypothesis testing, the test statistic (e.g. F, t, r) with confidence intervals, effect sizes, degrees of freedom and P value noted<br>*Give P values as exact values whenever suitable.*
☒	☐	For Bayesian analysis, information on the choice of priors and Markov chain Monte Carlo settings
☒	☐	For hierarchical and complex designs, identification of the appropriate level for tests and full reporting of outcomes
☐	☒	Estimates of effect sizes (e.g. Cohen's d, Pearson's r), indicating how they were calculated

Our web collection on statistics for biologists contains articles on many of the points above.

Software and code

Policy information about availability of computer code

Data collection	No software was used.
Data analysis	For modeling and criticality algorithms were written in Fortran and R (Rocha et. al. Sci. Rep. 2018). Lesions were segmented with Analyze 6.0; fMRI data were processed with in-house software; cortical surface reconstruction with Connectome Workbench; DWI preprocessing with Explore DTI; spherical deconvolution with Startrack; individual streamlines were registered with Track Queries; dissections with Trackvis.

For manuscripts utilizing custom algorithms or software that are central to the research but not yet described in published literature, software must be made available to editors and reviewers. We strongly encourage code deposition in a community repository (e.g. GitHub). See the Nature Portfolio guidelines for submitting code & software for further information.

Data

Policy information about availability of data

All manuscripts must include a data availability statement. This statement should provide the following information, where applicable:

- Accession codes, unique identifiers, or web links for publicly available datasets
- A description of any restrictions on data availability
- For clinical datasets or third party data, please ensure that the statement adheres to our policy

Source data are provided with this paper. Data to replicate all the figures and tables, as well as individual structural connectivity matrices for controls and patients have been deposited in Github (https://github.com/CorbettaLab/Rodrigo2022NatComm) and Zenodo (https://doi.org/10.5281/zenodo.6459955). Raw neuroimaging and neuropsychological data from Siegel et al (PNAS, 2016) and Corbetta et al (Neuron, 2015) are publicly available at cnda.wustl.edu and require controlled access as they contain sensitive patients' data. The person requesting the data must sign a confidentiality agreement provided by Washington University

stipulating that they will make no attempt to identify the patients and to use data only for research purposes. Correspondence and requests for materials should be addressed to R.P.R. (rodrigo.rocha@ufsc.br)

Field-specific reporting

Please select the one below that is the best fit for your research. If you are not sure, read the appropriate sections before making your selection.

☒ Life sciences ☐ Behavioural & social sciences ☐ Ecological, evolutionary & environmental sciences

For a reference copy of the document with all sections, see nature.com/documents/nr-reporting-summary-flat.pdf

Life sciences study design

All studies must disclose on these points even when the disclosure is negative.

Sample size	All data came from a large prospective longitudinal stroke study described in previous publications. The dataset includes 132 stroke patients at the sub-acute stage (2 weeks post-stroke). We used data from the subset of 103 patients who returned for clinical and imaging assessments at three monhts post-stroke, as well as the data from the 88 patients who returned for 1 year post-stroke assessment. The control group, formed by 28 individuals, was matched with the stroke sample for age, gender, and years of education. The sample size depended on the possibility to enroll as many stroke patients within the 5-year period of a NH grant.
Data exclusions	The inclusion/exclusion criteria were as follows: first symptomatic stroke, ischemic or hemorrhagic, clinical evidence of any neurological deficit. Exclusion: multiple lesions; Longstreth > 5 white matter disease; life expectancy < 1 yr; other neurological or psychiatric disorders.
Replication	The within-subject longitudinal design and double session in healthy controls allows to look at issues of replication. In previous work (e.g. Siegel et al PNAS 2016) we have looked at replication issues for the fMRI data; the DWI data were analyzed here for the first time.
Randomization	Subjects were either stroke or control subjects. There was no randomization.
Blinding	The two groups were not blind being clinical patients or controls. The analysis was set up to discriminate differences between groups, not to classify blindly one group vs. another.

Reporting for specific materials, systems and methods

We require information from authors about some types of materials, experimental systems and methods used in many studies. Here, indicate whether each material, system or method listed is relevant to your study. If you are not sure if a list item applies to your research, read the appropriate section before selecting a response.

Materials & experimental systems

n/a	Involved in the study	
☒	☐	Antibodies
☒	☐	Eukaryotic cell lines
☒	☐	Palaeontology and archaeology
☒	☐	Animals and other organisms
☐	☒	Human research participants
☐	☒	Clinical data
☒	☐	Dual use research of concern

Methods

n/a	Involved in the study	
☒	☐	ChIP-seq
☒	☐	Flow cytometry
☐	☒	MRI-based neuroimaging

Human research participants

Policy information about studies involving human research participants

Population characteristics	The dataset includes 132 stroke patients (mean age 54, standard deviation 11, range 19-83; 71 males; 68 left side lesions) at the sub-acute stage (2 weeks post-stroke). We used data from the subset of 103 patients who returned for clinical and imaging assessments at three monhts post-stroke, as well as the data from the 88 patients who returned for 1 year post-stroke assessment. The control group, formed by 28 individuals, was matched with the stroke sample for age, gender, and years of education. Data was collected twice in the healthy controls, 3 months apart.
Recruitment	Stroke patients were recruited prospectively through human studies committee approved rules and a dedicated stroke enrollment team.
Ethics oversight	Stroke patients and healthy controls provided informed consent as approved by the Washington University Institutional Review Board Behavioral assessment.

Note that full information on the approval of the study protocol must also be provided in the manuscript.

Clinical data

Policy information about clinical studies

All manuscripts should comply with the ICMJE guidelines for publication of clinical research and a completed CONSORT checklist must be included with all submissions.

Clinical trial registration	N/A
Study protocol	N/A
Data collection	This group of healthy and stroke participants began enrollment in a prospective stroke study at Washington University in 2010 with completion in 2015 (WU Stroke cohort I).
Outcomes	Patients were studied at three time points with a neurobehavioral battery and structural (T1/T2, Flair), functional (resting state), diffusion, and perfusion-based magnetic resonance imaging (MRI). Data was collected twice in the healthy controls, 3 months apart.

Magnetic resonance imaging

Experimental design

Design type	Data analysis and Modeling of longitudinal prospective neuroimaging data
Design specifications	Each subject (stroke or control) was studied at 3 time points: 2 weeks, 3 months, 12 months post-stroke.
Behavioral performance measures	A broad and in-depth neuropsychological battery including measures of attention, motor, memory, language, vision function was given to each subjects (Corbetta et al, Neuron 2015).

Acquisition

Imaging type(s)	MRI
Field strength	3T
Sequence & imaging parameters	Patients were studied 2 weeks (mean=13.4, SD=4.8 d), 3 months (mean=112.5 d, SD=18.4 d), and 1 year (mean=393.5 d, SD=55.1 d) post-stroke. Diffusion data were obtained only at 3 months and 1 year. Controls were studied twice with an interval of 3 months. All imaging was performed using a Siemens 3T Tim-Trio scanner at WUSM and the standard 12-channel head coil. The MRI protocol included structural, functional, pulsed arterial spin labeling (PASL) and diffusion tensor scans. Structural scans included: (i) a sagittal T1-weighted MPRAGE (TR=1,950 ms, TE=2.26 ms, flip angle=90°, voxel size= 1.0x1.0x1.0 mm); (ii) a transverse T2-weighted turbo spin echo (TR=2,500 ms, TE=435 ms, voxel size=1.0 x 1.0 x 1.0 mm); and (iii) sagital fluid attenuated inversion recovery (FLAIR) (TR=7,500 ms, TE=326 ms, voxel size 1.5 x 1.5 x 1.5 mm). PASL acquisition parameters were: TR=2,600 ms, TE=13 ms, flip angle= 90°, bandwith 2.232 KHz/Px, and FoV 220 mm; 120 volumes were acquired (322 s total), each containing 15 slices with slice thickness 6 - and 23.7 mm gap. Resting state functional scans were acquired with a gradient echo EPI sequence (TR=2,000 ms, TE=27 ms, 32 contiguous 4- mm slices, 4x4 mm in-plane resolution) during which participants were instructed to fixate on a small cross in a low luminance environment. Six to eight resting state fMRI runs, each including 128 volumes (30 min total), were acquired. fMRI Data Preprocessing of fMRI data included: (i) compensation for asynchonous slice acquisition using sinc interpolation; (ii) elimination of odd/even slice intensity differences resulting from interleaved acquisition; (iii) whole brain intensity normalization to achieve a mode value of 1,000; (iv) removal of distortion using synthetic field map estimation and spatial realignment within and across fMRI runs; and (v) resampling to 3-mm cubic voxels in atlas space including realignment and atlas transformation in one resampling step. Cross-modal (e.g., T2 weighted to T1 weighted) image registration was accomplished by aligning image gradients. Cross-model image registration in patients was checked by comparing the optimized voxel similarity measure to the 97.5 percentile obtained in the control group. In some cases, structural images were substituted across sessions to improve the quality of registration
Area of acquisition	whole brain scan

Diffusion MRI ☒ Used ☐ Not used

Parameters: 60 directions and a single b-value of 1000 s/mm2

Preprocessing

Preprocessing software	For each slice, diffusion-weighted data were simultaneously registered and corrected for participant motion and geometrical distortion adjusting the diffusion directions accordingly (ExploreDTI http://www.exploredti.com). Spherical deconvolution was chosen to estimate multiple orientations in voxels containing different populations of crossing fibres. The damped version of the Richardson-Lucy algorithm for spherical deconvolution was calculated using Startrack (https://www.mr-startrack.com). Algorithm parameters were chosen as previously descrived. A fixed fibre response corresponding to a shape factor of alpha=1.5 10^-3 mm2/s was chosen. Fibre orientation distribution estimates were obtained by selecting the orientation corresponding to the peaks (local maxima) of the fibre orientation distribution (FOD) profiles. To exclude spurious local maxima, we applied both an absolute and a relative threshold on the FOD amplitude. A first absolute threshold was used to exclude intrinsically small local maxima due to noise or isotropic tissue. This threshold was set to 3 times the mean amplitude of a spherical FOD obtained from a grey matter isotropic voxel (and therefore also higher than an isotropic voxel in

	the cerebrospinal fluid). A second relative thrshold of 10% of the maximum amplitude of the FOD was applied to remove the remaining local maxima with values higher than the absolute threshold.
Normalization	Normalization to the MNI152 space was performed after reconstructing the streamline in the native space of the patients. We co-registered the structural connectome data to the standard MNI 2 mm space.
Normalization template	MNI152 space template.
Noise and artifact removal	To exclude spurious local maxima, we applied both an absolute and a relative threshold on the FOD amplitude. A first absolute threshold was used to exclude intrinsically small local maxima due to noise or isotropic tissue. This threshold was set to 3 times the mean amplitude of a spherical FOD obtained from a grey matter isotropic voxel (and therefore also higher than an isotropic voxel in the cerebrospinal fluid). A second relative thrshold of 10% of the maximum amplitude of the FOD was applied to remove the remaining local maxima with values higher than the absolute threshold.
Volume censoring	Individual streamline density volumes were registered to the streamline density template in the MNI152 space template masking for the lesion size and the same transformation was applied to the individual whole-brain streamline tractography using the trackmath tool distributed with the software package Tract Querier. Here uniform deformation was applied to the whole brain and did not produce distortion that mostly occur when applying T1w normalisation to tractography. Further quality of the streamline normalisation was visually inspected by an anatomist (MTS).

Statistical modeling & inference

Model type and settings	N/A
Effect(s) tested	N/A

Specify type of analysis: ☒ Whole brain ☐ ROI-based ☐ Both

Statistic type for inference (See Eklund et al. 2016)	N/A
Correction	N/A

Models & analysis

n/a	Involved in the study
☐	☒ Functional and/or effective connectivity
☐	☒ Graph analysis
☐	☒ Multivariate modeling or predictive analysis

Functional and/or effective connectivity	Functional connectivity based on Pearson correlation
Graph analysis	Binarized structural brain networks (SC); modularity; global efficiency; average degree
Multivariate modeling and predictive analysis	In our multivariate approach features of the individual SC matrices extracted by Principal Component Analysis (PCA) were used as multivariate predictors for a Ridge Regression (RR) model trained to predict patients criticality values. All predictors (PC scores) and the outcome variable (criticality value) were z-normalized before applying RR. All RR models were trained and tested using a leave-one-(patient)-out cross validation (LOOCV) loop.
